# Supplementary material for: Modeling the Health Impact and Cost-Effectiveness of a Combined Schoolgirl HPV Vaccination and Cervical Cancer Screening Program in Guangdong Province, China
Source: Children (Basel). 2024 Jan 15;11(1):103. doi: 10.3390/children11010103 (PMC10814869; doi:10.3390/children11010103)
Supplement: Supplementary file 1 [file children-11-00103-s001.zip › children-2785024-supplementary.pdf]

Supplementary Materials

I. Model overview

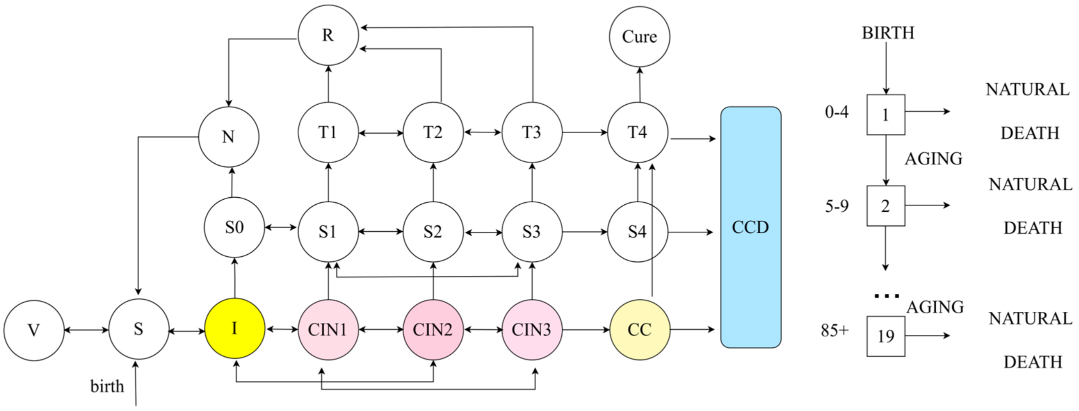

V: vaccine protected; S: susceptible; I: infected with HPV; P: infected with HPV and seroconverted; N: cleared of HPV; R: recovered from treatment for cervical intraepithelial neoplasia (CIN)1-3; CIN1-3: developed Cervical Intraepithelial Neoplasia 1-3; CC: developed cervical cancer; S0-S4: diagnosed as HPV DNA positive, CIN1-3, cervical cancer with cervical cancer screening; T0-T4: accepted treatment after diagnosis; Cure: Cured from cervical cancer by treatment.

Figure S1. Model diagram

II. Epidemiological and behavioral parameters

Table S1 Epidemiological parameters

Indicators	Symbol	Value	Reference
Spontaneous seek for cervical cancer treatment	*sp*	0.97 (0.95 - 1.00)	[1]
Seroclearance rate of HPV	σ	0.761 (0.624 - 0.820)	[2]
Seroconversion rate of HPV	τ	0.359 (0.318 - 0.609)	[3]
Natural clearance of HPV	c		[3, 4]
10-29 years		0.63(0.593-0.7952)	
30-49 years		0.38(0.286-0.419)	
>50 years		0.1004(0.055-0.1567)	
Progression rate from HPV infection to CIN1 in 12 months	$\rho_{1,1}$	0.238 (0.07 - 0.308)	[5]
Progression rate from CIN1 to CIN2 in 12 months	$\rho_{1,2}$	0.224 (0.161 – 0.298)	[6]

Progression rate from CIN2 to CIN3 in 12 months	$\rho_{1,3}$	0.350 (0.017 - 0.101)	[6]
Progression rate from HPV infection to CIN2 in 12 months	$\rho_{1,4}$	0.012 (0.008 - 0.17)	[7]
Progression rate from CIN1 to CIN3 in 12 months	$\rho_{1,5}$	0.046 (0.0098 – 0.129)	[6, 8, 9]
Progression rate from CIN3 to cervical cancer in 12 months	$\rho_{1,6}$	0.005(0.002-0.008)	[4]
Regression rate from CIN1 to HPV infection in 12 months	$\gamma_{1,1}$	0.495 (0.465 – 0.565)	[4]
Regression rate from CIN2 to CIN1 in 12 months	$\gamma_{1,2}$	0.249 (0.199 – 0.299)	[4, 6]
Regression rate from CIN3 to CIN2 in 12 months	$\gamma_{1,3}$	0.001 (0 - 0.002)	[4, 6]
Regression rate from CIN2 to HPV infection in 12 months	$\gamma_{1,4}$	0.190(0.1425-0.2375)	[4, 6]
Regression rate from CIN3 to CIN1 in 12 months	$\gamma_{1,5}$	0.100 (0 - 0.079)	[4, 6]
Cervical cancer death rate after treatment	δ_2	0.025(0.025 - 0.045)	[1]
Proportion of CIN1 accept treatment	o_1	0.457 (0.457 - 0.457)	[10]
Proportion of CIN2/3, CC accept treatment	$o_{2,3,4,5}$	1.00 (0.95 - 1.00)	[10]
Curative rate of CIN1 treatment in 12 months	t_1	0.98 (0.98 - 0.98)	[11]
Curative rate of CIN2 treatment in 12 months	t_2	0.924 (0.867 - 0.98)	[12, 13]
Curative rate of CIN3 treatment in 12 months	t_3	0.836 (0.688 - 0.983)	[12, 13]
Curative rate of cervical cancer treatment in 12 months	t_4	0.446 (0.26 - 0.631)	[14]
HPV clearance rate after CIN treatment in 12 months	c	0.805 (0.664 - 0.957)	[15]

Table S2 Intervention and behavioral parameters

**Indicators**	**Symbol**	**Value**	**Reference**
Cervical screening coverage in 3 years	sc	0.318	[16]
Sensitivity of HPV DNA screening	ss_0	0.925 (0.9 - 0.95)	[17, 18]
Sensitivity of primary screening	ss_1	0.715(0.48 - 0.95)	[17, 18]
Sensitivity of secondary screening	ss_2	0.93(0.923 - 0.936)	[19]
Women accept HPV DNA screening (35-64)	sa_0	0.755(0.755 - 0.755)	[18]

Women accept cytological screening (35-64)	sa_1	0.869(0.869 - 0.869)	[18]

$$se_0 = \left(\frac{sc}{3}\right) \cdot ss_0 \cdot sa_0$$

$$se_1 = \left(\frac{sc}{3}\right) \cdot ss_1 \cdot ss_2 \cdot sa_1$$

Parameter se_0 denotes the screening effectiveness for HPV positivity with the DNA test. Parameter se_1 denotes the screening effectiveness for CIN1-3 and cervical cancer with cytological or histological examinations.

Cytology and HPV testing-based are two commonly used methods for the cervical cancer screening, and HPV testing-based is increasingly recommended as the primary screening methods. Most guidelines recommended five years interval for the HPV testing-based screening or co-testing (HPV testing and cytology) based screening. For managing population with abnormal cervical cancer screening, triage or screening repeatedly to identify high-risk populations were more recommended. Direct colposcopy or treatment were allowed for women with higher risk of cervical intraepithelial neoplasia (CIN) during the screening procedure. According to the Chinese accelerated elimination of cervical cancer action plan, screening coverage should reach 50% in women aged 35–64 years by 2025, reach 70% by 2030. We simplify the screening to primary and secondary screenings in the model. For the primary screening, women aged 35-64 years are recommended to conduct cervical cancer screening using HPV DNA test and cytological tests. Secondary screening is based on histological examination with colposcopy or biopsy, which is performed to confirm the results of primary screening. Women showed positive signs in primary screening are advised to

accept secondary screening for confirmation. 3-year screening coverage *sc* is divided by five to obtain screening coverage in one year. We multiplied the one-year screening coverage (*sc*) with screening sensitivities (*ss*) and then adjusted it with screening age coverage (*sa*) to obtain se_0 and se_1.

III. Model equations

Following equations show the linked system with ordinary differential equations. This system tracks the population number change in each compartment.

$$\frac{dS}{dt} = \pi + \sigma_v V + \sigma N + cI - \eta_\omega S - \lambda IS - \mu S + AS$$

$$\frac{dV}{dt} = -\sigma_v V + \eta_\omega S - \mu V + AV$$

$$\frac{dI}{dt} = \lambda IS + \gamma_{1,1} CIN1 + \gamma_{1,4} CIN2 - cI - \rho_{1,1} I - \rho_{1,4} I - se_0 I - \mu I + AI$$

$$\frac{dCIN1}{dt} = \rho_{1,1} I + \gamma_{1.2} CIN2 + \gamma_{1.5} CIN3 - se_1 CIN1 - \gamma_{1,1} CIN1 - \rho_{1,2} CIN1 - \rho_{1,5} CIN1 - \mu CIN1 + ACIN1$$

$$\frac{dCIN2}{dt} = \rho_{1,2} CIN1 + \gamma_{1,3} CIN3 + \rho_{1,4} I - se_1 CIN2 - \gamma_{1,2} CIN2 - \gamma_{1,4} CIN2 - \rho_{1,3} CIN2 - \mu CIN2 + ACIN2$$

$$\frac{dCIN3}{dt} = \rho_{1,3} CIN2 + \rho_{1,5} CIN1 - se_1 CIN3 - \gamma_{1,3} CIN3 - \gamma_{1.5} CIN3 - \rho_{1,6} CIN3 - \mu CIN3 + ACIN3$$

$$\frac{dCC}{dt} = \rho_{1,6} CIN3 - pCC - \delta_1 CC - \mu CC + ACC$$

$$\frac{dS0}{dt} = se_0 I + \gamma_{11} S1 - \rho_{1,1} \tau_h S0 - c_h S0 - \mu S0 + AS0$$

$$\frac{dS1}{dt} = se_1 CIN1 + \rho_{1,1} \tau_h S0 + \gamma_{1.2} S2 + \gamma_{1.5} S3 + \rho_{1,1}(1 - c_1)R - o_1 S1 - \gamma_{1,1} S1 - \rho_{1,2} S1 - \rho_{1,5} S1 - \mu S1 + AS1$$

$$\frac{dS2}{dt} = se_1 CIN2 + \rho_{1,2} S1 + \gamma_{1,3} S3 - o_2 S2 - \gamma_{1,2} S2 - \rho_{1,3} S2 - \mu S2 + AS2$$

$$\frac{dS3}{dt} = se_1 CIN3 + \rho_{1,3} S2 + \rho_{1,5} S1 - o_3 S3 - \gamma_{1,3} S3 - \rho_{1,6} S3 - \mu S3 + AS3$$

$$\frac{dS4}{dt} = \rho_{1,6}S3 - o_4 S4 - \delta_1 S4 - \mu S4 + AS4$$

$$\frac{dT1}{dt} = o_1 S1 + \gamma_{2,1}T2 - t_1 T1 - \rho_{2,1}T1 - \mu T1 + AT1$$

$$\frac{dT2}{dt} = o_2 S2 + \rho_{2,1}T1 + \gamma_{3,2}T3 - t_2 T2 - \gamma_{2,1}T2 - \rho_{2,2}T2 - \mu T2 + AT2$$

$$\frac{dT3}{dt} = o_3 S3 + \rho_{2,2}T2 - t_3 T3 - \gamma_{3,2}T3 - \rho_{2,3}T3 - \mu T3 + AT3$$

$$\frac{dT4}{dt} = pCC + \rho_{2,3}T3 + o_4 S4 - t4T4 - \delta_2 T4 - \mu T4 + AT4$$

$$\frac{dR}{dt} = t_1 T1 + t_2 T2 + t_3 T3 - \left(c_1 + \rho_{1,1}(1 - c_1)\right)\mathrm{R} - \mu R + AR$$

$$\frac{dN}{dt} = c_h \mathrm{S0} + c_1 R - \sigma_h N - \mu N + AN$$

$$\frac{dCure}{dt} = t4T4 - \mu Cure + ACure$$

$$\frac{dD}{dt} = \delta_1 CC + \delta_1 S4 + \delta_2 T4$$

IV. Model calibration

The data of HPV prevalence used to calibrate the model were obtained from the epidemiological studies [20, 21]. The incidence and mortality rate of cervical cancer were retrieved from the 2010–2016 cancer registry data in Guangdong as reported by the Guangdong Cancer registry.

Table S3 Calibration data, 2010-2016

Year	Incidence rate of cervical cancer (/10^5)	Mortality rate of cervical cancer (/10^5)
2010	13.95	3.76
2011	12.51	3.12
2012	14.39	3.12
2013	14.00	3.61
2014	13.50	3.97
2015	13.84	4.34
2016	15.28	4.57

V. Cost-effective analysis

1. Medical expenditure

Cervical cancer screening cost, treatment cost (CIN1/CIN2/CIN3/cervical cancer) and, vaccination cost are three components included in medical expenditure. The price of different types of medical services is shown in Table S3. Most of the price values referred to research papers by CE Levin et al. published in 2011 and 2015. These prices were adjusted by the currency exchanging rate between CNY and USD in the reported year and 2023.

Table S4. Medical expenditure

Service type	Method	Price (US $)	Reference
HPV DNA screening	Hybrid Capture 2 HPV-DNA test	8.70	[22]
Primary cervical screening	visual inspection with acetic acid/ liquid-based cytology/ conventional cytology	9.57	[22]
Secondary cervical screening	Colposcopy/biopsy	14.60	[23, 24]
Indirect medical cost	Transportation expenses, Charge for loss of working time	16	[25]
CIN1 treatment	LEEP/ CKC	59	[23, 24]
CIN2 treatment	LEEP/ CKC	145.39	[23, 24]
CIN3 treatment	Hysterectomy/ LEEP/ CKC	786.02	[23, 24]
Cervical cancer treatment	Simple Hysterectomy and Chemotherapy	3405.04	[23, 24]

2. DALY calculation

The intervention effectiveness is represented by Disability-Adjusted Life-Year (DALY). By taking the burden from multiple HPV induced morbidities into account, DALY measures the gap between current health status and ideal health status of the entire population. DALY is composed of Years of Life Lost (YLL) due to premature mortality in the population, and the Years Lost due to Disability (YLD). We calculate the DALY on a yearly basis using the following equations:

$$DALY = YLL + YLD$$

$YLL = N \times L$

N: Number of deaths

L: Life expectancy of general women - the age when patient die due to cervical cancer.

$YLD = P \times DW$[26, 27]

P: number of prevalent cases of CIN1, CIN2, CIN3, cervical cancer

DW: Disability Weight

Table S5. Disability Weight

**Indication**	**Disability Weight**
CIN1	0
CIN2	0.1238
CIN3	0.1941
Cervical cancer	0.3071

3. Incremental cost-effective ratio (ICER)

The ICER is an essential index to assess the cost-effectiveness of interventions. Explicitly, the 'average cost per DALY averted' in 78 years was defined as ICER in this study. Total cost and the accumulated number of cervical cancers, cervical cancer death cases, and DALYs in each intervening scenario were noted in Table 2 and Table 3.

$$ICER = \frac{COST_2 - COST_1}{DALY_1 - DALY_2}$$

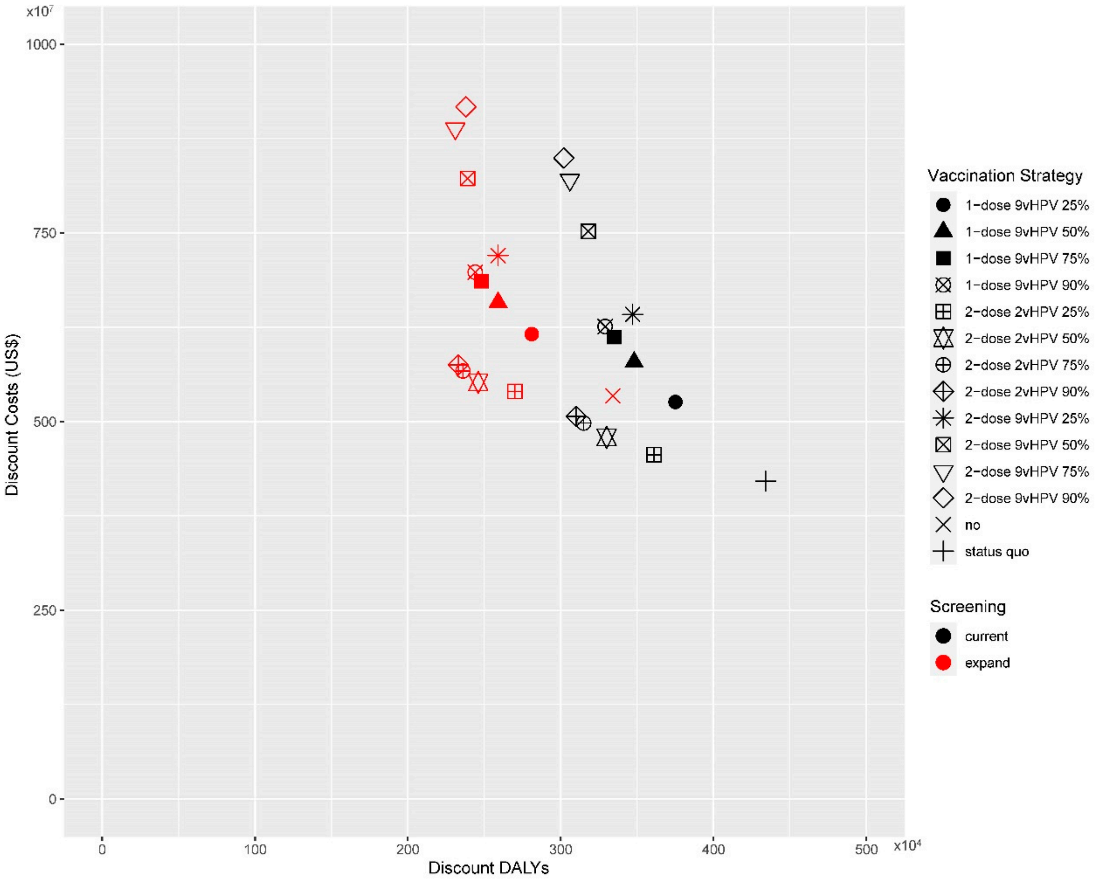

Figure S2. Cost-effectiveness plane for different strategies

References

1. Ma X, Harripersaud K, Smith K, Fairley C K, Zou H, Zou Z, Wang Y, Zhuang G, Zhang L. Modeling the epidemiological impact and cost-effectiveness of a combined schoolgirl HPV vaccination and cervical cancer screening program among Chinese women. Hum Vaccin Immunother. 2021, 17(4):1073-1082. doi:10.1080/21645515.2020.1832835.
2. Schmeink C E, Massuger L F A G, Lenselink C H, Quint W G V, Witte B I, Berkhof J, Melchers W J G, Bekkers R L M. Prospective follow-up of 2,065 young unscreened women to study human papillomavirus incidence and clearance. Int J Cancer. 2013, 133(1):172-181. doi:10.1002/ijc.27986.
3. Ermel A C, Shew M L, Weaver B A, Qadadri B, Denski C, Tu W, Tong Y, Fortenberry J D, Brown D R. DNA detection and seroprevalence of human papillomavirus in a cohort of adolescent women. Sex Transm Infect. 2014, 90(1):64-69. doi:10.1136/sextrans-2012-050886.
4. Xia C, Hu S, Xu X, Zhao X, Qiao Y, Broutet N, Canfell K, Hutubessy R, Zhao F. Projections up to 2100 and a budget optimisation strategy towards cervical cancer elimination in China: a modelling study. Lancet Public Health. 2019, 4(9):e462-e472. doi:10.1016/S2468-2667(19)30162-8.

5. Siebert U, Sroczynski G, Hillemanns P, Engel J, Stabenow R, Stegmaier C, Voigt K, Gibis B, Hölzel D, Goldie S J. The German cervical cancer screening model: development and validation of a decision-analytic model for cervical cancer screening in Germany. Eur J Public Health. 2006, 16(2):185-192. doi:10.1093/eurpub/cki163.
6. Haeussler K, Hout A v d, Baio G. A dynamic Bayesian Markov model for health economic evaluations of interventions in infectious disease. BMC Med Res Methodol. 2018, 18(1):82. doi:10.1186/s12874-018-0541-7.
7. Termrungruanglert W, Havanond P, Khemapech N, Lertmaharit S, Pongpanich S, Khorprasert C, Taneepanichskul S. Cost and effectiveness evaluation of prophylactic HPV vaccine in developing countries. Value Health. 2012, 1 Suppl(15):S29-34. doi:10.1016/j.jval.2011.11.007.
8. Xia C, Xu X, Zhao X, Hu S, Qiao Y, Zhang Y, Hutubessy R, Basu P, Broutet N, Jit M, et al. Effectiveness and cost-effectiveness of eliminating cervical cancer through a tailored optimal pathway: a modeling study. BMC Med. 2021, 19(1):62. doi:10.1186/s12916-021-01930-9.
9. Liu Y, Zhang Q, Hu S, Zhao F. Effect of vaccination age on cost-effectiveness of human papillomavirus vaccination against cervical cancer in China. BMC Cancer. 2016(16):164. doi:10.1186/s12885-016-2207-3.
10. Lv W. Cervical Intraepithelial Neoplasias (CIN) Clinical Treatment Guideline (ASCCP). Zhejiang Province gynaecology Annual Conference; 2008; Zhejiang Province. 2008. 2008:38-43.
11. Shi K, ZHAO S, JIANG L, LI X. Clinical analysis of application of chitosan gynecological biological dressing or Yunnan Baiyao after low-grade cervical intraepithelial neoplasia CO2 laser treatment. Chinese Journal of Obstetrics and Gynecology. 2016(5):401-403.
12. Sun L, Cao D, Yang J, Bian M, Wei L, Yang L, Cheng N, Yang Y, Wang Y, Cheng X, et al. The medical implication of cervical ring resection and CO2 laser vaporization for cervical intraepithelial neoplasia grade 2. Medical Journal of Peking Union Medical College Hospital. 2011, 2(2):129-133. doi:10.3969/j.issn.1674-9081.2011.02.008.
13. Wang Y, Kong W, Wu Y, Zhang W. Effect of Cervical Cold Cone Coneectomy and Total Hysterectomy on Treatment Outcomes of CINIII Combined with High-risk HPV Positive. Journal of Practical Obstetrics and Gynecology. 2016(02):122-125.
14. Li X. A Study on the Trend of Inpatients' Treatment Effect and Medical Cost of Female Cancer in Ten Years in Lanzhou. Lanzhou: Lanzhou University; 2008.
15. Ma G, Wang B, Ding H. Application of photodynamic combined therapy in the treatment of condyloma acuminatum. Chinese Journal of AIDS & STD. 2014(2):105-108.

16. Yuanyuan X. Study on the Influencing Factors of Cervical Cancer Mortality. Southern Medical University; 2021.
17. Catarino R, Schäfer S, Vassilakos P, Petignat P, Arbyn M. Accuracy of combinations of visual inspection using acetic acid or lugol iodine to detect cervical precancer: a meta-analysis. BJOG. 2019, 125(5):545-553. doi:10.1111/1471-0528.14783.
18. Guo R. Screening Methods of Cervical Cancer and Control Conditions of Screening Age. Journal of Clinical Medical Literature. 2015, 2(33):6841-6842.
19. Wang S, Xie D. The value of cervical fluid-based thin-layer cytology combined with colposcopy in the screening of cervical precancerous lesions. The Chinese Journal of Human Sexuality. 2015(11):36-39.
20. Jing L, Zhong X, Zhong Z, Huang W, Liu Y, Yang G, Zhang X, Zou J, Jing C, Wei X. Prevalence of human papillomavirus infection in Guangdong Province, China: a population-based survey of 78,355 women. Sex Transm Dis. 2014, 41(12):732-738. doi:10.1097/OLQ.0000000000000201.
21. Zeyan Z, Hua Z, Xingming Z, Xiaoping Z, Zhulin M, Yongxia W, Xiangcai W, Ling Y. Investigation on HPV infection status in women of childbearing age among floating population in Guangdong province. Contemporary Medicine. 2014, 20(09):30-31. doi:10.7620/zgfybj.j.issn.1001-4411.2013.28.38.
22. People's Government of Guangdong Province. Notice on Issuance of the Implementation Plan of the "Two Cancers" Free Examination Program for urban and rural Women in Guangdong Province (2020-2022). 2021 May 6. [Accessed 2023 July 1].
23. Levin C E, Sharma M, Olson Z, Verguet S, Shi J, Wang S, Qiao Y, Jamison D T, Kim J J. An extended cost-effectiveness analysis of publicly financed HPV vaccination to prevent cervical cancer in China. Vaccine. 2015, 33(24):2830-2841. doi:10.1016/j.vaccine.2015.02.052.
24. Levin C E, Sellors J, Shi J, Ma L, Qiao Y, Ortendahl J, O'Shea M K H, Goldie S J. Cost-effectiveness analysis of cervical cancer prevention based on a rapid human papillomavirus screening test in a high-risk region of China. Int J Cancer. 2010, 127(6):1404-1411. doi:10.1002/ijc.25150.
25. Yang Q, Liu D, Lou P, Kong Y, Dong Z, Zhang P, Chen P, Chang G, Dong D. Results and cost-effectiveness analysis of lung cancer screening for urban residents in Xuzhou from 2014 to 2019. Chinese Journal of Cancer Prevention and Treatment. 2022, 29(7):463-467.
26. Salomon J A, Vos T, Hogan D R, Gagnon M, Naghavi M, Mokdad A, Begum N, Shah R, Karyana M, Kosen S, et al. Common values in assessing health outcomes from disease and injury: disability weights measurement study for the Global Burden of Disease Study 2010. Lancet (London, England). 2012, 380(9859):2129-2143. doi:10.1016/S0140-6736(12)61680-8.
27. 1 C J L M, Theo Vos R L, Naghavi M, Flaxman A D, Michaud C, Ezzati M, Shibuya K, Salomon J A, Abdalla S, Aboyans V, et al. Disability-adjusted life

years (DALYs) for 291 diseases and injuries in 21 regions, 1990-2010: a systematic analysis for the Global Burden of Disease Study 2010. Lancet. 2012, 380(9859):2197-2223. doi:10.1016/S0140-6736(12)61689-4.
